# The global neuronal workspace as a broadcasting network

**DOI:** 10.1162/netn_a_00261

**Published:** 2022-10-01

**Authors:** Abel Wajnerman Paz

**Affiliations:** Department of Philosophy, Universidad Alberto Hurtado, Santiago, Chile; Neuroethics Buenos Aires, Buenos Aires, Argentina

**Keywords:** Consciousness, Global neuronal workspace, Broadcasting, Functional connectivity

## Abstract

A new strategy for moving forward in the characterization of the global neuronal workspace (GNW) is proposed. According to Dehaene, Changeux, and colleagues (Dehaene, 2014, pp. 304, 312; Dehaene & Changeux, 2004, 2005), broadcasting is the main function of the GNW. However, the dynamic network properties described by recent graph theoretic GNW models are consistent with many large-scale communication processes that are different from broadcasting. We propose to apply a different graph theoretic approach, originally developed for optimizing information dissemination in communication networks, which can be used to identify the pattern of frequency and phase-specific directed functional connections that the GNW would exhibit only if it were a broadcasting network.

## INTRODUCTION

Dehaene, Changeux, and colleagues postulate the existence of a global network or a “global neuronal workspace” (GNW) constituted by a set of cortical neurons that send projections to many distant areas through long-range excitatory axons. The main function of this network is to make the information encoded in a given specialized processor globally available by broadcasting it to all brain systems, a process that constitutes the neural basis of access to consciousness (Dehaene, 2014, pp. 304, 312; Dehaene & Changeux, 2004, 2005).

Although the model has been supported through the detection of key neural signatures associated with GNW broadcasting, these are not substantially different from those that could be associated with alternative large-scale processes. Perhaps the more precise characterization of these signatures has been provided by recent models describing graph theoretic properties that were found in transient undirected functional GNW networks. These properties indicate a high degree of “integration” between network components and therefore contribute to moving forward in our understanding of the connection between GNW signatures and broadcasting. Nevertheless, integration only entails efficient communication between GNW nodes and is therefore consistent with very different communication processes. By contrast, a GNW broadcasting model entails dynamic network properties uniquely tied to broadcasting. The next section characterizes the mentioned ambiguity of the GNW model. We then present a framework that can be used to depict a set of neural signatures exclusively associated with a GNW broadcasting process and a possible approach to experimentally detect them. A GNW broadcasting scheme is constituted by a specific pattern of frequency and phase-specific directed functional connections that could be detected through the application of phase transfer entropy (PTE) to the EEG signals that pick up the GNW’s “ignition.”

## GNW SIGNATURES

### The Four Original Signatures

According to the workspace model, the GNW breaks the modularity of the cortex by making the information encoded within any given specialized (and otherwise encapsulated) processor globally available, that is, by broadcasting it to all the other processors (Dehaene & Changeux, 2004). This broadcasting process was originally associated with four predicted neural “signatures,” that is, neural markers that reliably indicate that the stimulus was consciously perceived.

The first two signatures describe, respectively, the spatial and temporal properties of a large-scale activity pattern that characterizes conscious states. First, conscious perception is an “avalanche” in which signals pick up strength as they progress forward into the cortex and are finally spread throughout parietal and prefrontal lobes, resulting in a sustained large-scale ignition reaching and connecting distant processors (Dehaene, 2014, pp. 223–225). The second signature characterizes the temporal properties of the conscious avalanche. Only for conscious stimuli, a late (300 ms after stimulus onset) slow wave of activity is amplified and flows into the prefrontal cortex and many other associative regions, and then back to visual areas (Dehaene, 2014, pp. 334, 335). Finally, two additional signatures provide a more precise characterization of the GNW global activity pattern: the active units exhibit high-frequency (gamma-band) oscillations and a massive long-distance phase synchrony between them (Dehaene, 2014, pp. 216, 262; Dehaene & Naccache, 2001; Mashour, Roelfsema, Changeux, & Dehaene, 2020).

These last two signatures are associated with the specific mechanism through which communication between GNW modules occurs. Dehaene suggests that the GNW implements Pascal Fries’s *communication through coherence* (CTC) mechanism (Dehaene, 2014, pp. 255 ff.; Fries, 2005, 2015). This is the proposal that gamma-band phase synchronization can have an essential role in communication between neural populations.

The basic idea is that rhythmic modulations of postsynaptic activity in a given neuron or set of neurons constitute rhythmic modulations in *synaptic input gain* or *excitability*. Presynaptic inputs that consistently arrive at moments of high postsynaptic input gain will be more effective than those arriving at random phases of the excitability cycle. When a postsynaptic neuronal group receives inputs from several different presynaptic groups, it will respond primarily to the presynaptic group to which it is coherent. Thus, effective communication requires rhythmic synchronization between pre- and postsynaptic neurons (Fries, 2005, 2009, 2015). This mechanism will be crucial for the discussion of our graph theoretic approach.

### Graph Theoretic Signatures

A key development in the characterization of GNW signatures comes from recent graph theoretic studies on dynamic functional brain networks. These explore the idea that cognitive tasks result from transient functional networks, established and dissolved on the timescale of milliseconds (Bola & Borchardt, 2016; Braun et al., 2015; Gonzalez-Castillo & Bandettini, 2018; Gonzalez-Castillo et al., 2012; Hutchison et al., 2013; Kucyi & Davis, 2014; Kucyi, Hove, Esterman, Hutchison, & Valera, 2017; Simony et al., 2016). Some of these studies characterize the GNW theory as implying such functional reorganization. These approaches offer a graph theoretic interpretation of the GNW’s ignition in terms of a transient functional network exhibiting forms of integration that maximize intermodular communication. I will mention three representative examples of this trend spanning the past decade.

Kitzbichler, Henson, Smith, Nathan, and Bullmore (2011) interpret the network organization predicted by workspace theory as a shift toward small-worldness in which the performance of tasks that require conscious access reduces minimum path length (maximizing integration) and reduces clustering or modularity (thus minimizing segregation). In turn, Godwin, Barry, and Marois (2015) argued that GNW ignition is associated with a degradation of modularity via an increase in the participation coefficient, that is, an increase in functional connectivity across modules rather than within modules. Finally, Deco, Vidaurre, and Kringelbach (2021) argue that GNW intermodular integration must be characterized through the concept of a functional rich club. During GNW global ignition, specialized modules tend to be more densely functionally connected among themselves than to other brain regions (for complementary GNW analyses, see also Finc et al., 2019; Finc et al., 2017; and Vatansever, Menon, Manktelow, Sahakian, & Stamatakis, 2015).

These findings constitute a crucial step toward a mechanistic understanding of the GNW. A key insight is that the large-scale communication between any given pair of GNW nodes depends not only on a mechanism involving those two nodes (such as CTC) but also on the *global* pattern of functional connections between *all* network nodes. That is, communication between any pair of GNW modules is facilitated by the transient functional connectivity of the whole network.

However, a key assumption of the GNW theory is underdetermined by the predictions provided by these network models. All the mentioned graph theoretic measures account for the “integration” of information by the GNW, which in this context is equivalent to a general notion of *communication*. Network properties such as reduced average path length, reduced modularity, and increased rich-club connectivity are used to indicate how communication between specialized modules is facilitated. In the same way as in the anatomical network models, these measures are employed in dynamic models to explain (following Sporns, Chialvo, Kaiser, & Hilgetag, 2004) how a network solves the trade-off between time and metabolic cost required for communication between a relevant set of nodes (Bullmore & Sporns, 2012; Chklovskii & Koulakov, 2004; Kaiser & Hilgetag, 2006; Sporns, 2016; Sterling & Laughlin, 2015, Chapter 13). Nevertheless, efficient communication is consistent with many different large-scale processes that may be different from broadcasting. This is why focusing on network properties uniquely tied to broadcasting is an appealing strategy for exploring the GNW further.

### Broadcasting Versus Alternative Communication Processes

**Figure 1.**
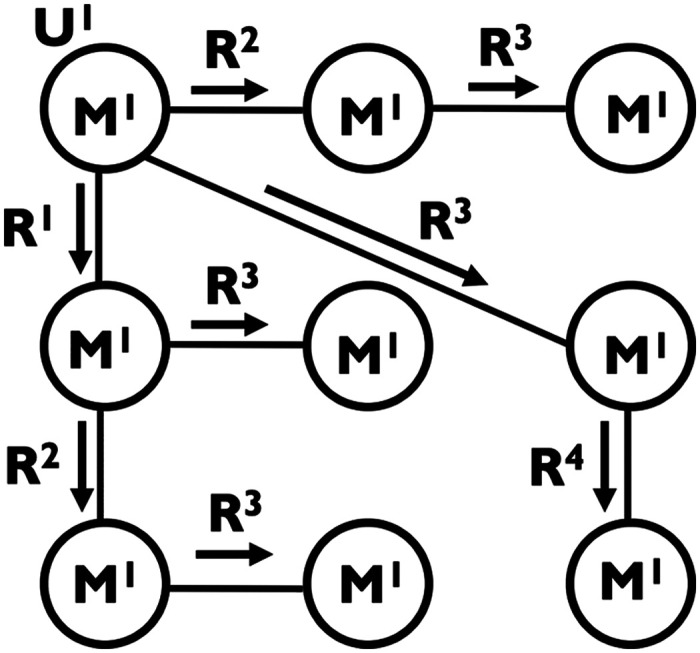
A communication network. Node U^1^ is the originator introducing message M^1^ to the network. Undirected edges represent communication lines between nodes. Directed edges represent the propagation of M^1^ from one node to another (i.e., a call) during one of the communication rounds R^1^–R^4^. In this case, the network is performing a broadcasting process.

A notion of broadcasting was developed within a graph theory research program that originated in the 1950s. It is focused on problems concerning information dissemination in communication networks with *multiple* sources and/or destinations (e.g., Bavelas, 1950; Landau, 1954; Shimbel, 1951). A communication network is presented as a graph *G* = (*V*, *E*) in which the set *V* of vertices or nodes corresponds to the members or processors of the network, and the set *E* of edges corresponds to the communication lines connecting pairs of members. A subset *U* of nodes are identified as the originators that introduce a set *M* of messages into *G*. During each communication round, each informed node makes a call (represented by a directed edge); that is, it sends a message to an uninformed node. During a series of rounds in which each node is either a message sender or a receiver, a communication task is completed (Figure 1).

**Figure 2.**
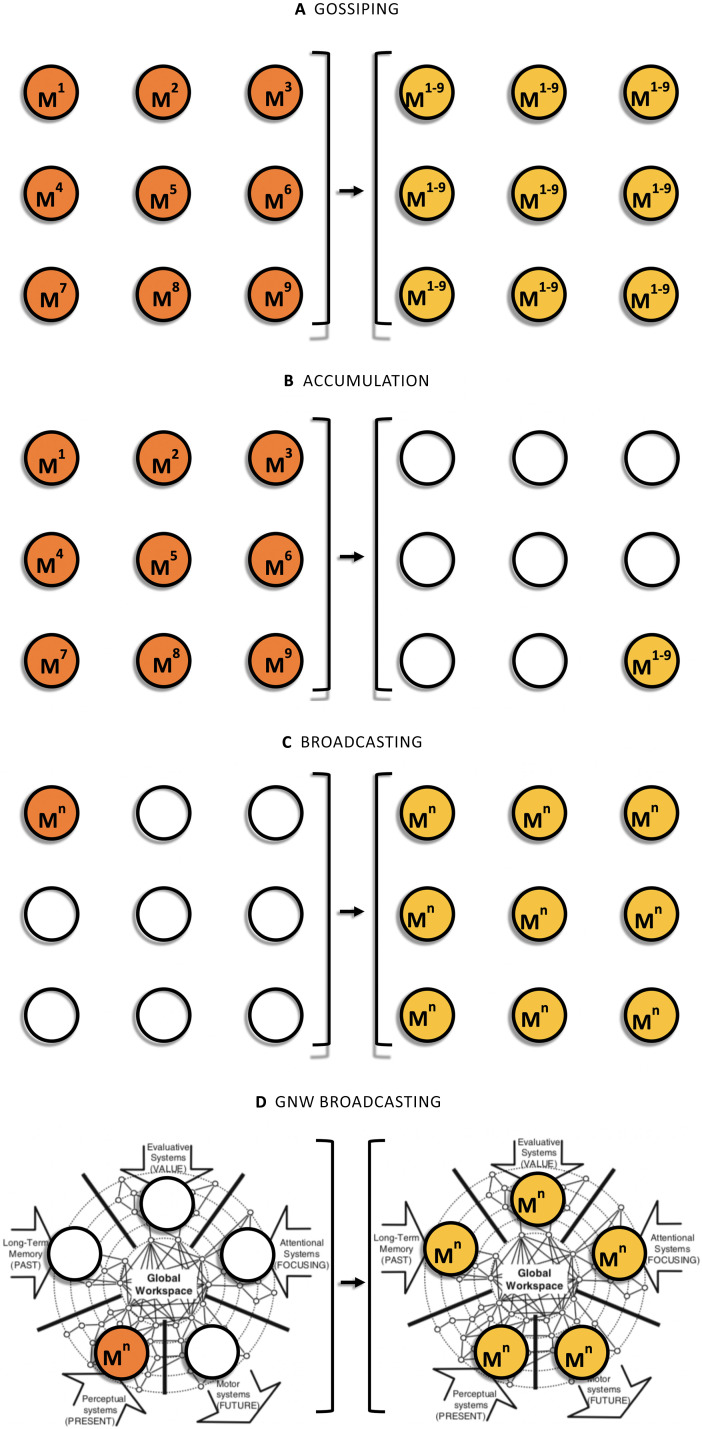
An input-output representation of three communication tasks: gossiping (A), accumulation (B), and broadcasting (C). Nodes on the left side represent the initial state of the network (the initial distribution of messages M^1^–M^n^), whereas nodes on the right side represent the result of the relevant communication algorithm. A GNW broadcasting model (D) can be used to determine what network properties the GNW would have if it were exclusively dedicated to solve this third problem.

For instance, Hajnal, Milner, and Szemeredi (1972) considered the so-called gossip problem, which can be characterized as follows: There is a scandal, which can be divided into *n* different pieces of information, and there are *n* people, each of whom knows one piece of scandal that is not known to any of the others. The problem is to determine how many calls are needed before all the people know all the scandal (Figure 2A). The accumulation problem is a second task. In this case, we have the same initial conditions but the task is to accumulate or send the *n* pieces of information from all the sources to a *single* receiver in the network (Hromkovič et al., 2005, p. 26; Figure 2B).

A major variant of the gossip problem is the broadcasting problem. Whereas gossiping is an all-to-all information dissemination process and accumulation is an all-to-one process, broadcasting is a one-to-all process. Broadcasting is the process in a communication network *G* = (*V*, *E*), whereby a message *m* (or set of messages *M*) originated by one root or source node *u* ∈ *V* is transmitted to all the nodes of the network (Hedetniemi, Hedetniemi, & Liestman, 1988; Figure 2C).

These tasks define different optimization problems that will have different solutions for a given number *n* of nodes. Therefore, if the GNW can be characterized as an efficient broadcasting system (Figure 2D), we should be able to identify signatures that are different from those it would exhibit if it were dedicated to an alternative communication process. In the next section the kinds implications that a broadcasting model entails will be articulated.

## TAKING BROADCASTING SERIOUSLY

### The Broadcast Problem

Broadcasting is accomplished by placing a series of “calls” over the communication lines of a network. According to the original version of the problem, the main goal is to complete this task as quickly as possible (for further discussion, see the section below entitled Neural Broadcasting Design Variables). In order to achieve this, a broadcast algorithm or scheme must be designed. A broadcasting scheme for a message *m* is the specification of a set of calls in a graph *G* originating from a vertex *u* to be made during successive time steps or rounds until all network nodes receive *m* (Farley, 2004). The broadcast scheme generates a broadcast tree, which is a spanning tree of the graph rooted at the originator (Harutyunyan, 2014; Harutyunyan & Li, 2017, 2020; Harutyunyan, Liestman, Peters, & Richards, 2013). The broadcast tree is simply the set of communication lines required to execute a given scheme.

The original formulation of the broadcast problem involved a set of restrictions for calls. These represent constraints imposed by some of the systems to which the framework was originally applied (e.g., people communicating by telephone). Therefore, they may have to be revised if we want to apply this approach to a brain system (see the section below entitled Neural Restrictions on the Broadcast Model). The original rules determined that (a) each call involves only two nodes (a sender and a receiver), (b) each call requires one round or unit of time, (c) a node can participate in only one call per unit of time, (d) a node can only call its neighbors (i.e., its adjacent nodes), and (e) many calls can be performed in parallel (e.g., Farley, Hedetniemi, Mitchell, & Proskurowski, 1979; Harutyunyan, 2014; Hedetniemi et al., 1988).

The basic broadcasting optimization problem is to find the scheme that minimizes the number of rounds required to complete broadcasting from a message originator, node *u*, in a connected graph with *n* nodes. The minimum time for broadcasting from *u* in a given graph *G* with *n* nodes is called the broadcast time *b* (*u*) of a vertex *u* in *G*. The task is to find the graph that can implement a scheme with minimal *b* (*u*), which is a *minimum broadcast tree* (a tree for which *b* (*u*) = ⌈log_2_*n*⌉ in networks constrained by the rules mentioned above; Proskurowski, 1981).

**Figure 3.**
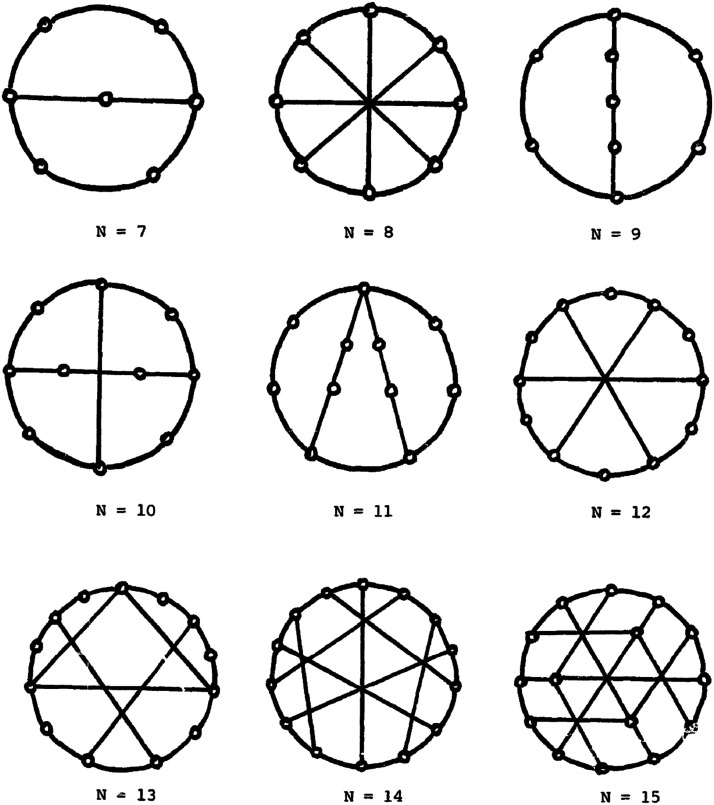
From Farley et al. (1979). Minimum broadcast graphs for *n* = 7–15 nodes.

A more complex version of this problem is to determine how efficient a network is in broadcasting from *any* of its nodes. The broadcast time of the whole graph *G*, *b* (*G*), is defined as equal to the maximum broadcast time of any vertex *u* in *G*, that is, *b* (*G*) = *max* {*b* (*u*) | *u* ∈ *V* (*G*)} (e.g., Harutyunyan & Li, 2017). In this case, the optimization problem is to find *n* schemes for broadcasting in an *n* node network, each of which determines a minimal broadcast tree with its root in a different node. The graph that results from combining these trees is a *broadcast graph*. The broadcast time of the graph *b* (*G*) seems a plausible design variable for the GNW. This is because all the specialized processors must be able to make their outputs globally accessible. Finally, efficient broadcasting may also be required to minimize wiring cost. The *minimum broadcast graph* (Figure 3) is a graph on *n* vertices with optimal *b* (*G*) and minimum number of edges, determined by a broadcast function *B* (*n*) (Farley, 1979; Harutyunyan & Li, 2017).

The present framework entails that if the GNW is an efficient broadcasting network connecting *n* nodes, it will exhibit very specific structural and functional properties (i.e., its broadcast graph and broadcast schemes, respectively).

### A Neural Broadcast Model

Characterizing the *specific* predictions that a GNW broadcasting model entails (i.e., the GNW’s broadcast graph and schemes) requires experimentally determining the value of some key network parameters (see below), and therefore it is beyond the scope of this perspective. However, this section and the two that follow will conceptualize the kinds of predictions that the framework can make (e.g., explain what a *GNW* broadcasting scheme is); identify the parameters that must be experimentally determined for computing the specific GNW broadcast graph and schemes; and propose a possible approach for experimentally testing these specific predictions.

The first step in the characterization of the GNW as a broadcast network is finding an adequate parcellation scheme. Given that the function of the GNW is to broadcast signals from any given specialized module to all the others, the natural choice is to characterize these modules as the nodes of the GNW broadcast network. Crucially, unlike nodes in alternative macroscale parcellation strategies (e.g., sensor-based schemes), modules may define actual anatomical and functional neural boundaries that can be detected through graph theoretic methods. In graph theoretic terms, a module (also called “community”) is a subset of nodes within a network that exhibit dense internal connections between them but weak or sparse connections with nodes that do not belong to that subset. These are often considered the building blocks in the organization of brain networks and are detected through different methods, of which the most widely applied is modularity maximization. This method aims to maximize a modularity quality function Q (Newman & Girvan, 2004), where a partition of a network into different communities has a high Q value when its communities are more internally dense than would be expected by chance (for a technical and methodological analysis of this approach, see Betzel, 2020; Sporns & Betzel, 2016).

This notion is different from the characterization of modules in cognitive science as systems specialized for realizing particular cognitive functions (Fodor, 1983), often defined by a set of special features such as informational encapsulation and inaccessibility, fast and mandatory processing, fixed neural architecture, and/or domain specificity, among others. As we saw, the GNW presupposes a modular architecture in this last sense: The GNW is supposed to diminish the modules’ informational encapsulation. However, the connection between graph theoretic and cognitive modules has been explored in both structural and functional brain networks. For instance, the community structure that was discovered in the *C. elegans* network through different methods (Bassett et al., 2010; Sohn, Choi, Ahn, Lee, & Jeong, 2011; Towlson, Vértes, Ahnert, Schafer, & Bullmore, 2013) seems to line up with the organization of its functionally specialized structures (e.g., Jarrell et al., 2012; Pan, Chatterjee, & Sinha, 2010; Sohn et al., 2011). Other examples of anatomical modules that map onto known cognitive modules include *Drosophila* (Shih et al., 2015), mouse (Wang, Sporns, & Burkhalter, 2012), and rat (Bota, Sporns, & Swanson, 2015) brain. In the human brain, Crossley et al. (2013) associated modules defined by functional connectivity with specific cognitive domains. More generally, it has been shown that functional modules identified through community detection methods line up with specialized modules with proprietary cognitive domains (Betzel, 2020; Meunier, Lambiotte, & Bullmore, 2010; Sporns & Betzel, 2016).

If the cognitive modules in Dehaene’s model also line up with community analysis, then its application as a parcellation scheme entails that the GNW has a relatively small number of nodes. This means that the task of finding the GNW broadcast graph and schemes is a relatively simple computational problem. The problem of finding the optimal broadcast algorithm for a network with an arbitrary number *n* of nodes is a hard problem (more precisely, an NP-complete problem; Farley et al., 1979; Garey & Johnson, 1979, Problem ND4). This is why minimum broadcast graphs have been determined for specific and relatively low values of *n* (see Figure 3). Global accessibility only involves perceptual, motor, long-term memory, evaluation, and attention systems (Dehaene & Naccache, 2001). By identifying GNW nodes with cognitive modules, we know that in this network *n* is low and its minimum broadcast graph is plausibly already known or easily determinable.

The next step is to understand how a broadcasting scheme (i.e., a sequence of calls) is accomplished between such set of nodes. We saw that a call is the process, represented by a directed edge, of sending a message from one node to another through a direct communication line, represented by an undirected edge. At the neural level, this could be understood as the propagation of an electrical (or electrochemical) signal from one neural structure to another through the fiber tract directly connecting them (Fornito, Zalesky, & Bullmore, 2016, Chapter 7). In network neuroscience terms, identifying signal propagation requires determining edge direction, which can be accomplished through different approaches, such as Granger causality (e.g., Deshpande, Santhanam, & Hu, 2011; Goebel, Roebroeck, Kim, & Formisano, 2003), dynamic causal modeling (e.g., Friston, Moran, & Seth, 2013; Kahan & Foltynie, 2013), and lagged correlations (Mitra & Raichle, 2016).

A call is not only defined by a directed functional connection between two nodes. It is also constituted by the specific communication line or anatomical edge underlying this functional connection. Calls bridge structural and functional connectivity. Different approaches are being developed for determining the relationship between functional and structural connections (e.g., Avena-Koenigsberger, Misic, & Sporns, 2018; Griffa et al., 2017; Mišić et al., 2016; Suárez et al., 2020; see Sadaghiani & Wirsich, 2020, for a review). Thus, a neural call will be a *directed functional connection between two nodes depending on a direct anatomical connection between them*. In turn, a broadcast scheme will be a *sequence* of such calls. That is, a scheme describes the trajectory or temporal pattern of signal propagation through a structural network.

Having identified the elements of neural broadcasting, we can now specify what kind of predictions the model will make regarding GNW structural and functional properties. A first prediction is that the anatomical connections between *n* GNW modules will resemble the broadcast graph for *n* nodes. Assuming that the GNW has the anatomical structure of a small-world network, the broadcast model would describe the pattern of long-range intermodular connections (those reducing average path length) that specifically facilitates broadcasting. A second prediction is related to how the GNW broadcasting schemes will shape dynamical functional connectivity. During its ignition, the GNW will exhibit a specific pattern of directed functional dependencies between its nodes, which will have the form of a minimum spanning tree with its root at the originator module. Finally, given that broadcasting is accomplished through neural *calls*, a further prediction is that each functional edge between GNW nodes will depend on a structural edge belonging to the GNW broadcast graph.

How can these predictions be experimentally assessed? Regarding the anatomical properties associated with the broadcasting model, a first possibility is to explore them by employing any of the different methods for identifying structural macroscopic connectivity (anatomically segregated brain regions connected by interregional pathways), including invasive (e.g., histological dissection and staining, degeneration methods, or axonal tracing) and noninvasive in vivo mapping (e.g., diffusion MRI and tractography). For instance, by applying white matter tractography to diffusion MRI data, we can produce a structural connectivity matrix, representing connectivity between GNW nodes.

However, these matrices describe only direct connections between regions, and identifying and characterizing indirect polysynaptic connections may be crucial for computing the optimal GNW broadcasting schemes that will be executed over its structural connections. For instance, we will see in the next section (Neural Restrictions on the Broadcast Model) that broadcasting rounds can probably be implemented in the GNW by the oscillation cycles of the CTC mechanism. These cycles determine the time window during which communication between a pair of directly connected pre- and postsynaptic neurons is possible. Thus, communication through a path with *n* synaptic crossings will require *n* broadcasting rounds. Given that directly connected regions are generally sparse (there are no white matter tracts between many pairs of regions), the optimal strategy minimizing GNW broadcasting time should probably be computed over a weighted structural matrix including information about the time costs of indirect connections.

In a recent study, Seguin, Tian, and Zalesky (2020) analyzed polysynaptic neural signaling by transforming structural connectivity matrices into communication matrices that quantified the efficiency of communication between indirectly as well as directly connected regions under different network communication models, defined by different kinds of schemes or algorithms. Interestingly, the assessment of communication efficiency relied on applying these different optimization strategies to matrices with different kinds connectivity weights that operationalize metabolic factors shaping large-scale signaling (Bullmore & Sporns, 2012; Fornito et al., 2016; Rubinov & Sporns, 2010). Efficient communication will privilege high-volume white matter projections that may enable fast and reliable signal propagation, connections with a lower number of synaptic crossings, and connections with less physical length. Following this approach, the optimal GNW broadcasting schemes can be computed for a weighted structural graph representing some of these parameters. Crucially, the binary weight representing the number of synaptic crossings of a given edge connecting two GNW nodes can be used to measure its time cost in terms of broadcasting rounds (see the next section).

In turn, the assessment of the functional properties described by the broadcasting model presents different challenges. Functional connectivity is very often measured from functional magnetic resonance imaging (fMRI) data that, having a spatial resolution of the order of some millimeters, can be employed for reliably mapping large-scale functional networks (Fox & Raichle, 2007; Gillebert & Mantini, 2013). However, despite a number of technical issues, the higher temporal resolution electroencephalography (EEG) or magnetoencephalography (MEG) makes them potentially better suited than fMRI to capture the dynamics of GNW broadcasting, which is characterized by functional connections that rely on the CTC mechanism, that is, on the phase alignment of oscillations with specific frequencies.

Perhaps the main technical issue related to EEG spatiotemporal mapping is that at each channel, the signal is the result of the contributions from an unknown number of different sources, including distant neural and nonneural sources (Lopes da Silva, 2013). Consequently, sensor-level data cannot provide the information required to identify the spatial origin, trajectory, and destination of a neural broadcasting call. This is why source modeling is necessary to resolve (to some degree) the ambiguity of sensor-level analysis (Baillet, 2017; Lopes da Silva, 2013; Michel et al., 2004; Stropahl, Bauer, Debener, & Bleichner, 2018). For instance, Liu, Farahibozorg, Porcaro, Wenderoth, and Mantini (2017) and Liu, Ganzetti, Wenderoth, and Mantini (2018) have recently proposed the use of independent component analysis (ICA), which performs a blind decomposition of different spatiotemporal patterns that are mixed in the data, assuming that these patterns are mutually and statistically independent in time or space. ICA identifies a number of independent components, each of which consists of a spatial map and an associated time course (Calhoun, Adali, Pearlson, & Pekar, 2001). The IC spatial map reveals brain regions that have a similar response pattern, and are therefore considered to be functionally connected (Brookes et al., 2011; Mantini et al., 2007).

However, we saw that GNW broadcasting schemes are constituted by *directed* functional connections that depend on the phase alignment of oscillations with specific frequencies. A number of very recent EEG-based network analyses use phase transfer entropy (PTE) for identifying phase-specific directed functional connectivity as part of the biomarkers of different psychiatric disorders. PTE was presented by Paluš and Stefanovska (2003) and evaluated by Lobier, Siebenhühner, Palva, and Palva (2014), and is a reformulation of Granger’s causality principle mentioned above (Granger, 1969; Wiener, 1956). Unlike other phase synchrony metrics (Rosenblum, Pikovsky, & Kurths, 1996; Stam, Nolte, & Daffertshofer, 2007; Vinck, Oostenveld, van Wingerden, Battaglia, & Pennartz, 2011), PTE allows identification of the direction of information flow. Unlike other directed functional connectivity metrics, it allows identification of frequency and phase-specific information flow. For instance, Hasanzadeh, Mohebbi, and Rostami (2020) used PTE to discover patterns of directed connectivity associated with major depressive disorder. In addition to local and global efficiency, they calculated node degree (number of links connected to a node) and node strength (the sum of link weights connected to a node), separately computing inward and outward links (in-degree, in-strength, and out-degree and out-strength, respectively). In turn, Ekhlasi, Nasrabadi, and Mohammadi (2021) investigated directed functional connections in attention-deficit/hyperactivity disorder (ADHD) patients with EEG by using PTE in each frequency band during an attentional task. Among other findings, they showed that the posterior to anterior pattern of connectivity commonly seen in the control group is disturbed in the ADHD patients in the theta band during visual tasks. Finally, Al-Ezzi, Al-Shargabi, Al-Shargie, and Al-Shargabi (2022) developed an EEG study of functional directed connectivity for assessing the severity of social anxiety disorder (SAD) in different patients. They identified the direction of functional connections by using partial directed coherence (PDC) at four frequency bands (delta, theta, alpha, and beta). PDC is a frequency-domain metric similar to PTE that is also based on the Granger causality approach. In addition to other network properties, they also used in-degree, in-strength, and out-degree and out-strength for assessing the severity of SAD.

Thus, PTE or PDC could constitute a possible approach for assessing the direction of EEG-detected functional connections in the GNW. The GNW model predicts an intense propagation or ignition of neural activity particularly toward the prefrontal and parietal cortex at 200 to 300 ms after stimulus onset on trials with conscious perception. This is a robust signature that can be detected through EEG independently of stimulus modality or paradigm used to manipulate consciousness (Mashour et al., 2020). Given a GNW ignition originated from a specific module *u*, we can examine whether the system implements a broadcasting process by determining whether the direction of each gamma-band functional connection between GNW modules during this process is consistent with the direction of the calls that constitute the GNW scheme for broadcasting from *u*.

However, computing the GNW broadcasting schemes with which PTE analysis will be matched may require introducing a number of biologically plausible constraints and parameters that were not considered in the more basic versions of the broadcast model. These constraints will be examined in the next section.

### Neural Restrictions on the Broadcast Model

Calls (and consequently schemes) are also defined by the restrictions of the original version of the broadcast problem, which specify how they work in some of the systems to which the framework was originally applied (e.g., communication by telephone). These constraints strongly shape the predictions of our network model. Thus, it is crucial to assess whether they apply to neural processing. In this section, we will focus on what we take to be the most problematic constraints on calls.

First, we have to assess the constraints prohibiting that a given node has simultaneous relations with *n* > 1 nodes. These are the conditions that a node can participate in only one call per round and that each call involves only two nodes.

Telesford, Simpson, Burdette, Hayasaka, and Laurienti (2011) have analyzed information flow in brain networks by following a characterization of different flow types provided by Borgatti (2005). There are at least two classification parameters that are relevant for neural communication. First, nodes can communicate with each other via transfer (i.e., the message remains at only one node at a time) or via replication (i.e., the message is copied at each node). If a system communicates through replication, we should determine whether information is duplicated at one node at a time (serial) or simultaneously duplicated at several nodes (parallel). Telesford et al. (2011) claim that the brain uses parallel duplication. This is implied by how signal propagation works in divergent connections (i.e., multiple synaptic outputs from a single source). Activation of multiple synapses from a single terminal occurs simultaneously (e.g., Shepherd, 2003, p. 10). A neuron can send signals simultaneously to different postsynaptic neurons and, consequently, through different neural paths.

Fortunately, broadcasting processes with one-to-many relations have been considered in the literature. There are two different approaches to this form of broadcasting. In “radio broadcasting,” each node makes simultaneous calls to all of its neighboring nodes. In broadcasting with “conference calls,” each node makes one call per round but each call can involve *n* ≥ 2 nodes. A question for further research is to determine which, if either, of these approaches would be suitable for modeling the GNW. (For instance, Telesford et al., 2011, affirms that the firing neuron typically activates approximately 30% of all synapses in a stochastic manner. This seems to favor conference calls, in which not all of the postsynaptic neurons would be activated.)

Second, we have to examine the rule that each call requires one unit of time. This requires determining first whether there is a GNW round. Although the idea that neural processes in general can be parsed into regular and functionally relevant time intervals seems implausible (Piccinini & Bahar, 2013), it is possible that the GNW is an exception.

The idea that the CTC mechanism underlies communication in the GNW suggests a candidate for a GNW round. As we saw, synchronization between pre- and postsynaptic neurons determines the time window in which effective communication between them is possible. CTC demands that information is only sent at moments of high input gain in the postsynaptic oscillation cycle. This cycle is a possible candidate for a GNW communication round because, as we saw, the network produces a large-scale synchrony between its active units. This suggests that all of the GNW active units have a regular and shared series of time windows in which communication between them can occur. (Recall that within this context neural synchrony refers to *phase* alignment.) The identification between broadcasting rounds and oscillation cycles is a possibility that could be experimentally and theoretically explored.

Assuming that these cycles do constitute GNW rounds, what about the condition that each call occurs in one round? It seems that this condition should be revised. As we suggested, many edges in the GNW network are probably polysynaptic paths connecting two processors and therefore communication between processors could take more than one round. A possible way to address the broadcasting problem in a network not satisfying this one-round condition is by using a weighted graph in which each weight represents the time cost (i.e., the number of rounds) of communicating through a given edge. We saw that binary weights have been used to represent the number of synaptic crossings of a given edge (Seguin et al., 2020). In a broadcasting model the same weights could stand for the number of rounds required for sending a message through a given edge. Thus, the tree representing an optimal GNW broadcast scheme would be a weighted minimum spanning tree. The algorithm for developing a minimum spanning tree in a weighted graph was developed by Prim (1957).

An additional key constraint that a broadcasting model of neural signal propagation should account for is related to recent discussions on neural routing. Routing involves the control of paths that information can take across a network. Given that physical networks have limited resources, the role of routing is to allocate signal paths in a way that optimizes relevant communication goals, such as those defining the broadcasting problem (i.e., time and wire minimization). In this sense, a scheme constituting the optimal solution to a given broadcasting problem represents an efficient routing strategy. However, we still need to assess whether it lines up with the general strategies that are plausibly implemented by neural communication.

Daniel Graham distinguishes three different routing models that have been employed in neuroscience (e.g., Graham, 2014; Graham & Rockmore, 2011). According to a message-switched routing model, each message is passed along in its entirety from node to node. Graham suggests that it is implausible that this strategy is implemented by brain networks because message-switched routing requires memory buffers to store messages in a queue in which they wait their turn to be passed along. In turn, in circuit-switched routing an exclusive path is established between the nodes that send and receive a given message. However, such systems are plausibly not implemented by the brain, because, among other reasons, it does not have the resources for the all-to-all connectivity that exclusive paths between each sender and each receiver would require. Finally, in packet switching routing (the scheme used on the internet), messages at a source are chopped into small packets and then reassembled at their destination. As Graham and Rockmore (2011) point out, packet switching has several appealing parallels with cortical signaling. They emphasize that this strategy entails (a) an ability to dynamically reroute traffic, as cortex does following lesion; (b) a capacity for different applications (e.g., email, http) to run concurrently on the same system, as distinct modalities and signaling systems do in cortex; and (c) an inherent hierarchy of the network protocol stack, which mirrors hierarchical organization within and across cortex.

How would a GNW scheme look if it performed broadcasting by using packet switching routing? There is a version of the broadcasting problem, first studied by Chinn, Hedetniemi, and Mitchell (1979) and Farley (1980), in which the broadcasted message at an originator node can be represented as being chopped into different sub-messages. Given that each sub-message is broadcasted to all network nodes, all sub-messages will be reunited at each destination to be assembled, as packet switching requires. *Multiple message broadcasting* is the process of multiple message dissemination in a communication network in which *n* messages, originated by one vertex, are transmitted to all vertices of the network (Harutyunyan, 2006). In this case, the optimization problem requires finding, for *m* nodes, the graph and scheme with the minimum number of time units necessary to broadcast *n* messages to all vertices from any given originator.

Additionally, the fact that GNW broadcasting depends on CTC could also contribute to understanding how routing may work in this system. In CTC models of visual processing, the feedforward propagation of signals is modulated by top-down signals. If CTC also controls signals within the GNW, then their propagation schemes would also be regulated by feedback signals from receptor units. Graham (2014) has pointed out that neural feedback from higher levels in a processing hierarchy could be a fundamental aspect of neural routing. The optimization of GNW schemes predicted by the broadcasting model could be the result of signal routing through the CTC mechanisms.

Finally, there is an additional restriction that did not affect the original broadcasting model but may nonetheless be required for its neural implementation. We need to assess whether, for each node, sending a message (or a number of messages) can be a function of a number of inputs defining a transmission threshold. Very often neural communication depends on the summation of presynaptic potentials in a shared post postsynaptic neuron within a time window (e.g., the kind of integration performed by simple cells in the visual cortex). This kind of restriction would obviously affect broadcasting schemes, as a given node would make a call (or a number of simultaneous calls) when (and only when) a given number of signals have arrived from other nodes. However, the fact that GNW communicates through the CTC mechanism suggests that its broadcasting scheme will possibly not involve a fixed or general input-output rule of this kind. Recall that CTC’s main function is to modulate *input gain or excitability*, thus making possible to route neural signals in a flexible way by affecting the sensitivity of a given node to specific input signals (Fries, 2015). In CTC communication, postsynaptic units can selectively modulate which presynaptic are effective in producing postsynaptic activation and which are not. Additionally, GNW feedback projections act as distributed routers through which signals can be amplified, sustained, and spread (Mashour et al., 2020), modulating the strength of the input signals themselves. This routing is plausibly a form of balanced amplification that depends not only on interareal excitatory feedback connections but also on intra-areal lateral inhibition, so that the facilitation of signal propagation between weakly connected areas does not undermine the stability of more strongly connected areas (Joglekar, Mejias, Yang, & Wang, 2018). These top-down routing mechanisms can be used to adapt input-output relations at each GNW node to fit an optimal broadcasting scheme.

### Neural Broadcasting Design Variables

In addition to constraints, we must also consider whether the design variables that define the broadcasting problem (time and wiring costs) also require adjustment or reinterpretation in order to represent plausible GNW demands.

The idea that brain networks evolved to solve the trade-off between wiring cost and processing speed can be traced back to Ramón y Cajal’s time and space conservation laws (Bullmore & Sporns, 2012; Chklovskii & Koulakov, 2004; Kaiser & Hilgetag, 2006; Sporns, 2016; Sterling & Laughlin, 2015, Chapter 13). In network neuroscience, small-world networks have been proposed as a possible solution to this trade-off. Regular clustering minimizes wiring cost, whereas short average path length produced by random long-range connections minimizes conduction delay, thus increasing the speed at which information can be exchanged. Thus, the broadcast approach can be considered a development of small-world GNW models in the following sense: If (as Kitzbichler et al., 2011, argue) the GNW exhibits a small-world structure, then the communication processes it performs are plausibly optimized for minimizing time and wiring cost. The broadcast model then shows how the optimization of those specific parameters would affect the pattern of intermodular connections of this small-world network if it were dedicated exclusively to broadcasting.

Another possible worry is related to a design variable that seems to be crucial to neural design, namely, *energy cost*. In very early studies of neural information transmission, it has been suggested that because the brain is one of the metabolically most active organs of the body (Sokoloff, 1989), optimizing neural processing would require a compromise between energy and informational efficiency (e.g., Levy & Baxter, 1996). For instance, a long-standing hypothesis affirms that the visual system optimizes information processing by implementing sparse coding, which basically consists of representing each environmental condition by using very few active units (Barlow, 1961). This is why it is reasonable to ask whether and how the demand for energy cost minimization shapes a broadcast network. Calls seem to be a key component of broadcasting energy cost. A GNW call is a signaling process, and neural signaling has been considered a major element in the brain’s energy budget (Attwell & Laughlin, 2001). Thus, it is plausible that the cost of a broadcasting process is at least partially determined by the total number of calls required by the implemented algorithm or scheme.

Nevertheless, once we identify the number of calls as one of the key elements for estimating broadcasting energy cost, it becomes clear why this variable has not been considered in the literature. The main reason is that this number is constant, that is, alternative algorithms for broadcasting to a given number of nodes require the same amount of calls. Although the possibility of having simultaneous calls makes broadcasting time much smaller, *n* − 1 calls are always required to broadcast in graphs with *n* nodes (Richards & Liestman, 1988). For broadcasting in *k*-uniform hypergraphs (the kind of graph required by conference calls) with *n* nodes, *n* − 1 / *k* − 1 calls will be required.

Of course, energy cost makes no difference regarding algorithm choice only if we assume that all calls have the same cost. However, we saw that this is plausibly not the case for the GNW. Many GNW edges may be polysynaptic paths that require more than one round to make a call. Part of the energy cost of a particular call may be given by the *n* consecutive synapses that a signal has to pass through in order to go from one processor to another. If *n* is different for different GNW edges, then the broadcasting scheme could be optimized by using only the cheapest paths. However, notice that an energy weight of this kind would be redundant. If these weights are determined by the number of synaptic crossings of a given path, they will be equal to the time weight mentioned in the previous section.

## CONCLUSION

The graph theoretic characterization of the GNW theory’s key assumption, that is, that the GNW is a broadcasting network, can contribute to the development of its model. It predicts fine-grained network properties that are uniquely tied to broadcasting. Unlike current GNW network models, which focus exclusively on undirected functional connectivity associated with efficient communication, the broadcast model entails signal propagation hypotheses characterized in terms of directed functional connectivity. GNW broadcasting schemes are constituted by frequency and phase-specific directed functional connections that could be detected through the application of phase transfer entropy (PTE) to the EEG signals that pick up the GNW’s ignition. The computation of these schemes requires experimentally determining time weights for each GNW path through the detection of polysynaptic connections and theoretically determining a communication strategy (e.g., multiple vs. single message broadcasting and radio broadcasting vs. conference calls). Finally, the model is not an alternative to but a development of previous ones in that it abstracts away from intramodular connectivity and explores the specific pattern of long-range intermodular connections described by small-world GNW models.

## ACKNOWLEDGMENTS

The author thanks researchers that discussed previous versions of this manuscript, including Nicolás Serrano, Sabrina Haimovici, Arleen Salles, Francisco Pereira, Juan Manuel Garrido, Rodolfo Aldea, Gabriel Reyes, Ignacio Cea, Mazviita Chirimuuta, Alfredo Vernazzani, Daniel Burnston, and Julieta Picasso Cazón.

## AUTHOR CONTRIBUTIONS

Abel Wajnerman Paz: Conceptualization; Investigation; Writing – original draft; Writing – review & editing.

## FUNDING INFORMATION

PI: Abel Wajnerman Paz, Funding Agency: Agencia Nacional de Investigación y Desarrollo, Award ID: FONDECYT INICIACIÓN 11220327.

PI: Juan Manuel Garrido, Funding Agency: Fondo Nacional de Desarrollo Científico y Tecnológico, Award ID: FONDECYT REGULAR 1210091.

PI: Francisco Pereira, Funding Agency: Agencia Nacional de Investigación y Desarrollo, Award ID: FONDECYT REGULAR 1200197.

## TECHNICAL TERMS

Broadcasting: A one-to-all process dissemination process in a communication network *G* = (*V*, *E*) in which a message *m* (or set of messages *M*) originated by one root node *u* ∈ *V* is transmitted to all nodes in *G*.
Communication network: A graph *G* = (*V*, *E*) representing information dissemination with *multiple* sources and/or destinations.
Round: The time unit used to measure calls. Whereas in some networks each call takes one round, in a weighted communication network a weight can represent the number of rounds required to make a call through a given edge.
Call: The process, represented by a directed edge, of sending a message from one informed node to an adjacent uniformed node through a direct communication line, represented by an undirected edge.
Communication task: Sending a set *M* of messages in a communication network *G* from a set of informed nodes U ⊆ V to a set of uniformed nodes by placing a series of calls over the communication lines of a network, represented by *G*’s edges.
Broadcast problem: An optimization problem that consists of finding the scheme that has *b* (*u*) in a graph *G* with *n* nodes.
Broadcast scheme: The specification of a set of calls in a graph *G* originating from a node *u* to be made during successive rounds until all network nodes receive a given message *m*.
Broadcast tree: A spanning tree of a graph *G* rooted at the source *u* of a given broadcast scheme *s* for *G* that includes only the set of communication lines required to execute *s*.
Broadcast graph problem: The optimization problem that requires finding *n* schemes for broadcasting in an *n* node network, each of which has its root in a different node and minimal *b* (*u*).
Broadcast time: The minimum time, *b* (*u*), for broadcasting from *u* in a given graph *G* with *n* nodes.
Minimum broadcast tree: A subgraph containing only the edges necessary to implement a broadcast scheme with minimal *b* (*u*).
Broadcast time of a graph *G*: The time equal to the maximum broadcast time of any source node *u* in *G*, that is, *b* (*G*) = *max* {*b* (*u*) | *u* ∈ *V* (*G*)}.
Broadcast graph: The graph that results from combining the minimum broadcast trees for the schemes that solve a broadcast graph problem.
Minimum broadcast graph: A graph on *n* nodes with optimal *b* (*G*) and the minimum number of edges, determined by a broadcast function *B* (*n*).
